# Dual-wavelength single-frequency laser emission in asymmetric coupled microdisks

**DOI:** 10.1038/srep38053

**Published:** 2016-12-01

**Authors:** Haotian Wang, Sheng Liu, Lin Chen, Deyuan Shen, Xiang Wu

**Affiliations:** 1 Shanghai Ultra-precision Optical Manufacturing Engineering Research Center, Key Lab for Micro and Nanophotonic Structures (Ministry of Education), and Department of Optical Science and Engineering, Fudan University, Shanghai 200433, China

## Abstract

The gain and loss in a microcavity laser play an important role for the modulation of laser spectrum. We show that dual-wavelength single mode lasing can be achieved in an asymmetric coupled system consisted of two size-mismatched microdisks. The amount of eigenmodes in this coupled-microdisk system is reduced relying on the Vernier effect. Then a single mode is selected to lase by controlling the gain branching in the supermodes. The supermodes are formed by the coupling between different transverse whispering-gallery modes (WGMs). When the gain/loss status between the two mirodisks is changed through selectively pumping process, the modulated gain branching for various supermodes leads to the switchable single-frequency laser emission. The results obtained in this work will provide the further understand for the spectral modulation mechanism in the coupled microcavity laser system.

In the past few decades, whispering-gallery-mode (WGM) microcavity lasers have attracted much attention due to their advantages of high Q factors, low thresholds and compact structures^1^,^2^,^3^. Since the size of a WGM microcavity is usually much larger than the resonant wavelengths, the small free spectral range (FSR) and the broad gain spectrum result in multi-longitudinal-mode lasing operation, which limits the WGM microlasers for many applications such as optical communication and ultra-sensitive optical sensors. To address this issue, the asymmetric coupled WGM microcavity structure, which usually consists of two size-mismatched microcavities, has been proposed to simplify the laser spectrum. Based on the Vernier effect, the coupled microcavity system has an enlarged FSR, which enables only one longitudinal mode within the gain spectrum to obtain the single-mode lasing^4^,^5^,^6^. Recently, controlling the gain and loss elements has been proved to be an alternative approach for the spectral modulation in microcavity laser systems^7^,^8^,^9^,^10^,^11^,^12^. This type of spectral modulation introduces a unique phenomenon, namely parity-time (PT) symmetry, which has been studied extensively in recent works including both physical fundamentals and practical applications^13^,^14^,^15^,^16^,^17^,^18^,^19^,^20^,^21^,^22^,^23^,^24^,^25^,^26^,^27^,^28^,^29^,^30^. The eigenfrequency spectrum of the PT symmetry system remains real even if the gain and loss are introduced. When the gain-loss contrast exceeds a certain value, the symmetry is broken and the spectrum becomes complex^13^,^14^. Based on the PT symmetry-breaking concept, some researchers have successfully achieved the single-mode lasing in WGM microcavities^25^,^27^. In these cases, above the PT symmetry-breaking threshold, the eigenfrequency splitting of the supermodes formed by two coupled modes will transform into the imaginary domain. As the imaginary parts of the eigenfrequencies indicate the amplifying or decaying of the optical field, the gain branching in the supermodes occurs. Since the essence of the PT symmetry breaking is the gain branching, the spectral modulation by manipulating the gain/loss status is also applicable to the asymmetric coupled microcavity system. In this article, we combine the manipulation for the gain /loss status with the spectral modulation based on the Vernier Effect to realize the single-mode lasing in an asymmetric coupled microdisk system. In this coupled system, the supermodes are formed by the coupling between the WGMs with different radial orders. The dual-wavelength single-frequency operation can be achieved by implementing different selectively pumping schemes. According to the results obtained in this work, it is possible to advance the study on the physical foundation of the spectral modulation in the microcavity laser system and also provide a useful guideline for the design of microcavity lasers.

## Results and Discussion

### Theoretical Analysis

The eigenfrequencies of the supermodes in coupled microdisks can be written as:


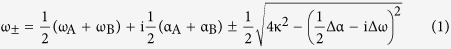


where Δω = ω_A_ − ω_B_ and Δα = α_A_ − α_B_, ω and α are the resonance frequency and gain/loss coefficient (α > 0 for gain and α < 0 for loss), respectively. The subscripts A and B indicate the different microdisks, respectively. κ is the coupling coefficient.

**Figure 1:**
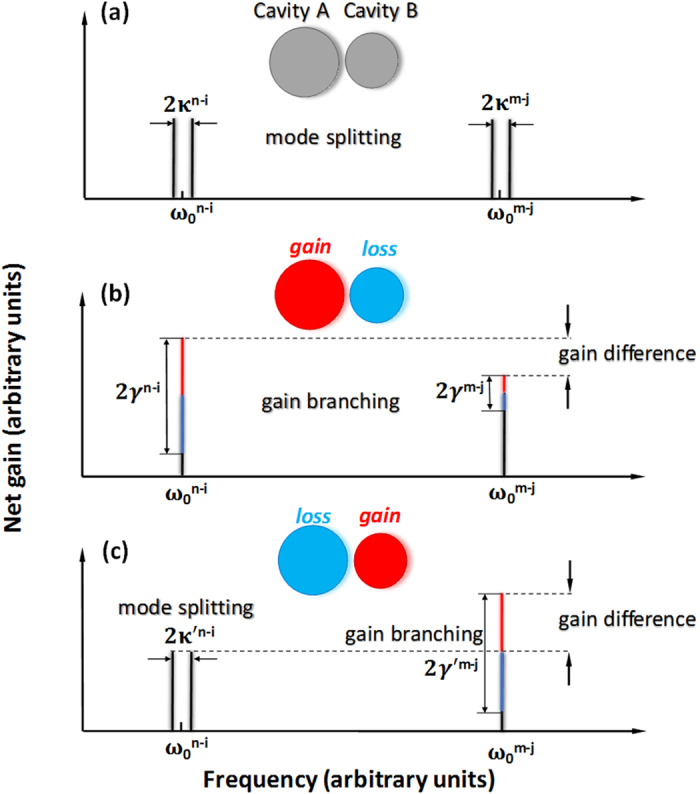
Schematic of the mode splitting and gain branching in the two coupled microdisks. (**a**) Mode splitting in the passive coupled microdisks. (**b**) and (**c**) Gain branching in the coupled microdisks with two different gain/loss status, respectively.

The Vernier effect is demonstrated schematically in Fig. 1(a) for two size-mismatched coupled-microdisks. To briefly illustrate this phenomenon, we take two different sets of WGMs in each microdisk as example. The letter m, n, i and j refer to the radial quantum number of the WGMs. The resonance frequency of the coupled system requires Δω = 0. For a conservative system, the gain or loss coefficient α are equal to zero. Thus, the Eq. (1) can be rewritten as:


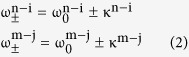


where 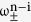 and 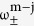 are the eigenfrequencies of the supermodes formed by the coupled modes n − i and m − j, respectively. For simplicity, we write n − i and m − j to indicate the coupling between the mode n and i, m and j, respectively. 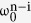 and 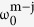 are the common resonance frequencies. Each pair of the supermodes have two eigenfrequencies, which is known as the mode splitting.

Now we think the above coupled cavities as an open system and introduce the gain/loss into the cavities. The frequency differences are originated from the third terms in Eq. (1). Note that the frequency splitting transit to imaginary domain when Δα^2^ >16κ^2^, now Eq. (2) becomes:


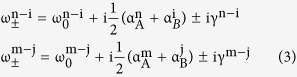


where we define the gain branching coefficients as γ and 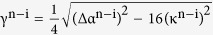 and 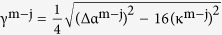, respectively. The positive imaginary part represents the amplification of the corresponding mode while the negative imaginary part means the suppression, which leads to the gain branching in the supermodes. To obtain a larger Δα that is enough to generate the gain branching, as shown in Fig. 1(b,c), the status where the gain in one cavity and loss in the other is introduced. In general, the different radial-order WGMs have different gain/loss coefficient α due to the different Q factors. Besides, in practical the mode-matching rate of the pump beam is not the same for different WGMs. As a result, the coupled modes n-i and m-j will have different Δα, which leads to the gain branching in different degrees. The difference between the gain branching enhances gain-difference of the two coupled modes then facilitates a single mode to lase. Figure 1(b,c) demonstrate the inverse gain/loss status in the coupled system. Exchanging the gain/loss status changes Δα as well, which modulates the gain branching in each coupled modes. As shown in Fig. 1(c), when Δα for the coupled mode n − i may be reduced to below the gain branching threshold, the spectral behavior of the coupled mode turns into mode splitting. On the contrary, the gain branching for the coupled mode m − j is enhanced and it becomes much easier to lase. Finally, a dual-wavelength single-frequency lasing can be achieved.

**Figure 2:**
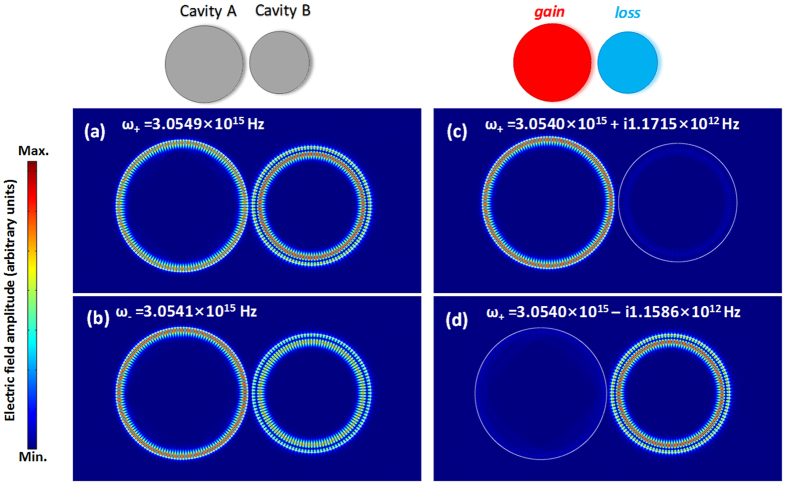
Calculated electric filed amplitude distributions and corresponding eigenfrequencies in two coupled microdisks. (**a,b**) The splitting of eigenfrequency in real domain in the passive microdisks. (**c,d**) The splitting of eigenfrequency transits to the imaginary domain when introduce different gain/loss status for the two microdisks.

Figure 2 shows the simulation results of the electric field distribution in an asymmetric coupled-microdisk system. In this case, the sizes of the two cavities are 10 and 9 μm, respectively. The gap between them is set as 400 nm. Figure 2(a,b) show the mode-splitting case when the gain/loss is not considered. The real parts of the eigenfrequencies diverge and the mode fields of the supermodes evenly distribute in two cavities. If the gain/loss is introduced into the cavities, the differences of the eigenfrequencies transit into imaginary domain contributing to the gain branching, which is shown in Fig. 2(c,d). In the case of the gain branching, the modes field predominantly reside in one of the microdisks. Consequently, the amplified modes have the positive imaginary parts and experience a net amount of gain. By comparison, the mode with the negative imaginary parts will be suppressed.

## Experimental Results

**Figure 3:**
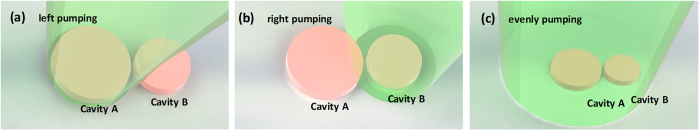
Schematic of three different pumping schemes.

To realize the different gain/loss status in the coupled microdisk system, three different pumping schemes are utilized as shown in Fig. 3. The “left pumping” scheme introduces the gain and loss into cavity A and B, respectively, while the “right pumping” scheme contributes a reverse gain/loss status. Note that the size of the pump beam spot is keep as constant in the two selectively pumping schemes. To do the control experiment, we expand the pump beam to let the two cavities are pumped simultaneously, which is indicated by “evenly pumping”.

**Figure 4:**
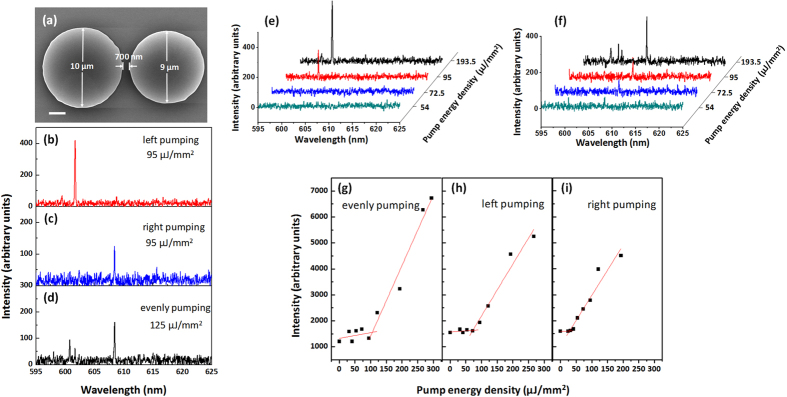
(**a**) Scanning electron microscope (SEM) images of the coupled microdisks. The scale bar is 2 μm. (**b,c**) The laser spectra under the three pumping schemes. (**e,f**) Laser spectra under different pump energy density for the “left pumping” and “right pumping” schemes, respectively. (**g–i**) Light-light curves for three pumping schemes.

Figure 4 demonstrates the laser spectra of the coupled microdisks when utilize different pumping schemes. Figure 4(a) is the scanning electron microscope (SEM) image of the coupled microdisks. The diameters of the microdisk cavities A and B are 10 and 9 μm, respectively. The gap between the them is around 700 nm. As shown in Fig. 4(b,c), the single-frequency laser is obtained at around 602 and 608 nm under the “left pumping” and “right pumping” schemes, respectively. Whereas, we do not find any single-frequency lasing in the “evenly pumping” scheme. As the above analysis, in the “evenly pumping” scheme, the gain-loss contrast between the two cavities Δα is not larger enough to generate the gain branching. Due to the intensive mode competition, a small gain-difference between the modes is not able to select a single mode to lase. Therefore, multi-mode lasing occurs once the pump intensity is beyond the laser threshold. When only one cavity is illuminated, Δα is sufficient to cause the gain branching. Different modes experience the gain branching in different degrees, then the gain-difference between the modes is enhanced. The mode corresponding to the largest degree of the gain branching is selected to lase. The gain/loss status between two cavities is exchanged when the pumping scheme is switched from “left pumping” to “right pumping”. It will readjust the degree of the gain branching for each mode. Another laser mode will be selected to lase resulting in the wavelength shift of the single-frequency lasing. Figure 4(e,f) show the laser spectra of the “left pumping” and “right pumping” schemes under different pump energy density, respectively. The difference of the pump energy density (Δp) between the emission of the single mode and the multimode emission is nearly 100 μJ/mm^2^ for the “left pumping” scheme, which is much larger than that of 20 μJ/mm^2^ for the “right pumping” scheme. It indicates that the mode lasing in the “left pumping” scheme has a larger degree of the gain branching, which is possible to provide a better side-mode suppression ratio (SMSR).

**Figure 5:**
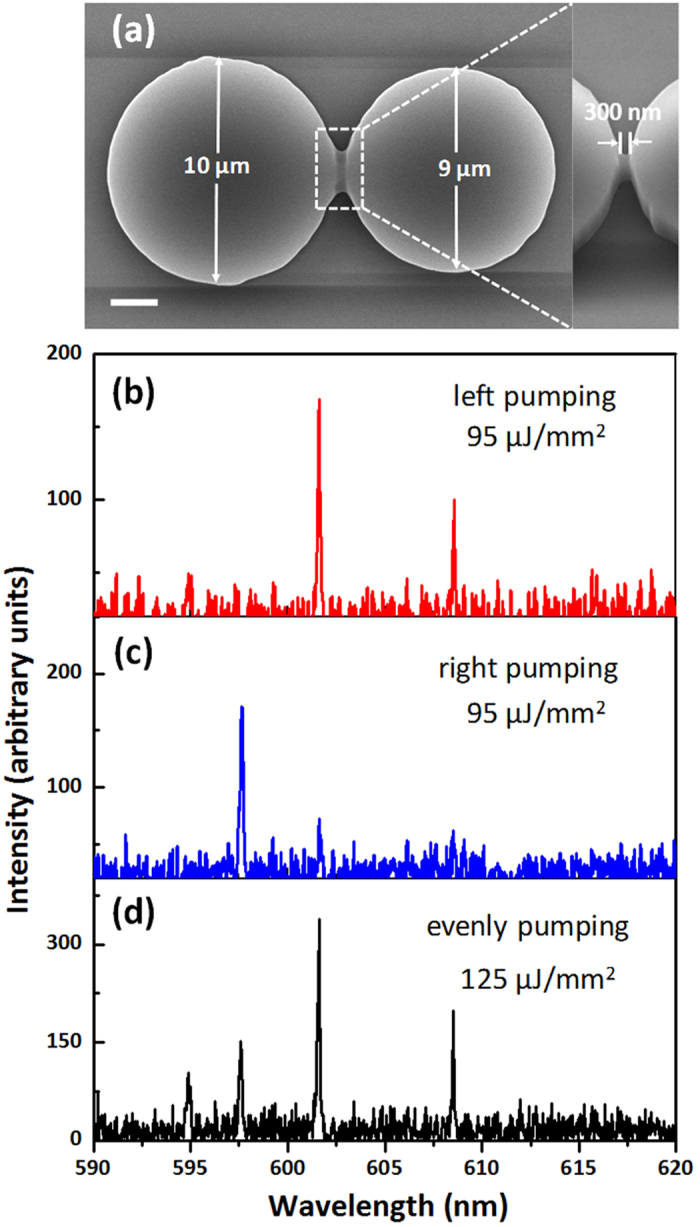
(**a**) Scanning electron microscope (SEM) images of the coupled microdisks with a smaller gap. The scale bar is 2 μm. (**b–d**) The laser spectra under three different pumping schemes.

The light-light curves for three pumping schemes is shown in Fig. 5. The slope of fitted lines for “evenly pumping” is larger than those for the “left pumping” and “right pumping” schemes. The different laser thresholds for the “left pumping” and “right pumping” schemes imply that the laser, which is lasing under different pump schemes, originates from different WGMs.

Figure 5 shows the laser spectra and the SEM image for the coupled microdisks with a smaller gap of 300 nm. In this case, the reduced gap increases the coupling coefficient κ. According to Eq. (4), it becomes more difficult to cause the gain branching to enhance the gain-difference between the laser modes. As expected, no single-frequency lasing has been observed even when the pump energy is down to the threshold.

**Figure 6:**
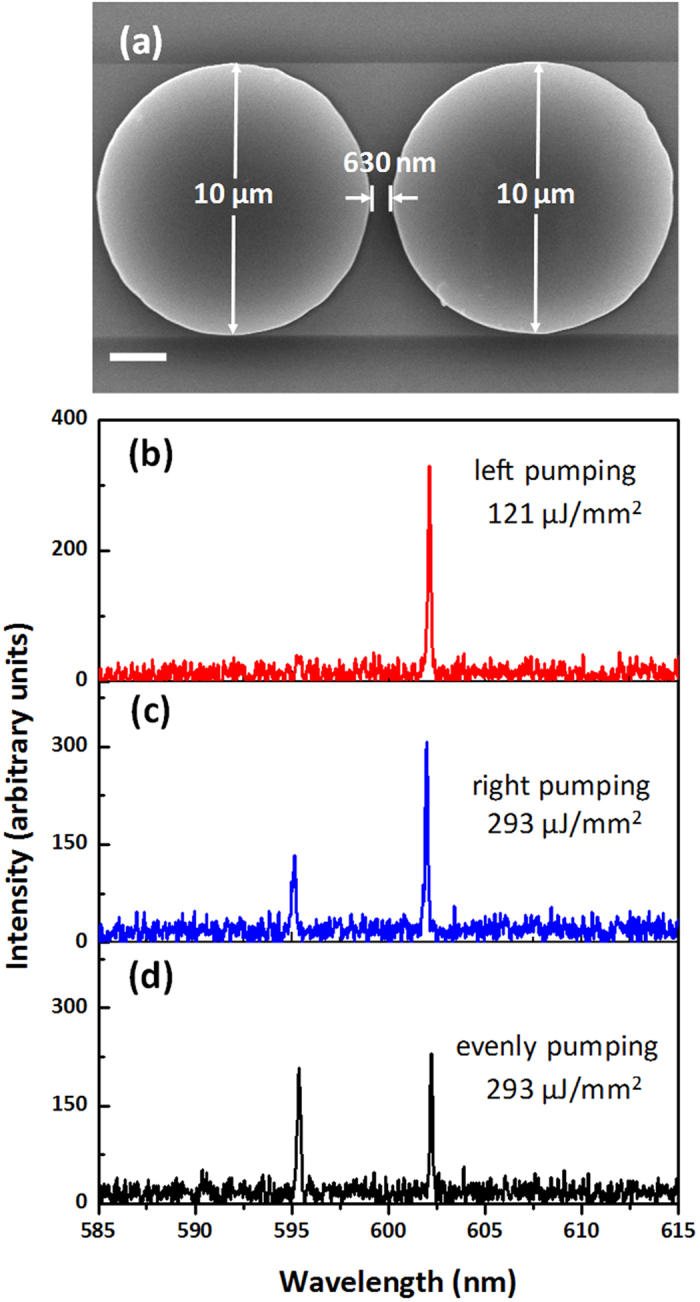
(**a**) Scanning electron microscope (SEM) images of two identical coupled microdisks of 10 µm with a 500 nm gap. The scale bar is 2 μm. (**b**) The laser spectra under three different pumping schemes.

In some symmetric coupled microcavity system such as identical coupled microrings^25^,^28^, the coupling usually occurs between the same transverse WGMs. Therefore, there is no difference in the lasing spectra when one single cavity is pumped selectively. However, in the coupled microdsik system, the high-order transverse WGMs could be excited and the coupling between different transverse WGMs may occur even in two identical microdisks. This asymmetric coupling in the symmetrical cavity structure leads to the different spectra when changes the pump scheme. Figure 6(a) shows the SEM image of two identical microdisks of 10 μm and the gap between them is about 630 nm. The laser spectra of this coupled microdisks under different pumping schemes are given in Fig. 6(b–d), respectively. The single-frequency lasing is only found in the “left pumping” scheme. These different spectra further explain that in the coupled microdisks the coupling will occur between different transverse WGMs.

## Conclusion

Based on the Vernier effect and the manipulation of the gain/loss status between the two cavities, we realize the single-frequency lasing in two size-mismatched coupled-microdisks. The different pumping schemes give rise to the single-frequency lasing at different wavelengths since the gain branching for each pairs of supermodes are modulated differently. The narrow gap between the microdisks makes it difficult to achieve the single-frequency lasing due to the weakened gain branching. In the coupled system consisted by two identical microdisk, the asymmetric laser spectra appear under a symmetric cavities structure when the pumping scheme is changed, which implies each pair of the supermodes are formed by the coupling between different radial-order WGMs. The results presented in this work will provide the method for realizing the wavelength-tunable on-chip microlaser under single-frequency operation.

## Method

### Numerical simulation

Our numerical simulation is based on the finite element method (FEM). The 2-dimensional (2D) model is adopted using the effective refractive index of 1.55 for the three-layer slab waveguide (Air- SU-8-SiO_2_). The gain/loss in the cavity could be realized by set the imaginary part of the refractive index of the cavity. In our simulation, the imaginary part of the refractive index value 0.0006 and −0.0006 are set as the gain and loss in the cavity, respectively. The surrounding media is air and the outermost shell of perfect match layer (PML) is used to absorb the outgoing waves.

### Sample fabrication

We use the Rhodamine B doped SU-8 as the active media. A 1 μm active layer was deposited by spin coating onto the silicon substrates with a 2 μm thick thermal oxide layer. The microdisk patterns were generated by putting the microcavity mask on top of the active layer and illuminating under a Karl–Suss MJB3 Mask Aligner.

### Experimental setup for selectively pumping Schemes

A frequency-doubled output (at 532 nm) of a Nd:YAG mode-locked laser is used as the pump laser. The pulse duration is 35 ps with a repetition rate of 10 Hz. The pump beam size is controlled by a tunable beam expanding system and the energy is controlled by a tunable attenuator. The beam spot in the microscope is further focused into nearly 10 microns by using a 50 × objective len. The sample of the coupled mircodisks is placed on a 3-D stage that is used to adjust the relative position between the cavities and the pump spot. The output laser is collected by an optical multi-channel fiber bundle (400-μm core diameter). The light from the fiber bundle is coupled to the input slit of an *f* = 0.75 m monochromator and detected by an EMCCD.

## Additional Information

**How to cite this article**: Wang, H. *et al*. Dual-wavelength single-frequency laser emission in asymmetric coupled microdisks. *Sci. Rep.* **6**, 38053; doi: 10.1038/srep38053 (2016).

**Publisher's note:** Springer Nature remains neutral with regard to jurisdictional claims in published maps and institutional affiliations.
